# Assessing Empathy across Childhood and Adolescence: Validation of the Empathy Questionnaire for Children and Adolescents (EmQue-CA)

**DOI:** 10.3389/fpsyg.2017.00870

**Published:** 2017-05-29

**Authors:** Sandy Overgaauw, Carolien Rieffe, Evelien Broekhof, Eveline A. Crone, Berna Güroğlu

**Affiliations:** ^1^Institute of Psychology, Leiden UniversityLeiden, Netherlands; ^2^Leiden Institute for Brain and CognitionLeiden, Netherlands

**Keywords:** empathy measure, prosocial motivation, validation questionnaire, bullying, friendship quality, emotion awareness, developmental changes

## Abstract

Empathy plays a crucial role in healthy social functioning and in maintaining positive social relationships. In this study, 1250 children and adolescents (10–15 year olds) completed the newly developed Empathy Questionnaire for Children and Adolescents (EmQue-CA) that was tested on reliability, construct validity, convergent validity, and concurrent validity. The EmQue-CA aims to assess empathy using the following scales: *affective empathy, cognitive empathy*, and *intention to comfort*. A Principal Components Analysis, which was directly tested with a Confirmatory Factor Analysis, confirmed the proposed three-factor model resulting in 14 final items. Reliability analyses demonstrated high internal consistency of the scales. Furthermore, the scales showed high convergent validity, as they were positively correlated with related scales of the Interpersonal Reactivity Index (Davis, 1983). With regard to concurrent validity, higher empathy was related to more attention to others’ emotions, higher friendship quality, less focus on own affective state, and lower levels of bullying behavior. Taken together, we show that the EmQue-CA is a reliable and valid instrument to measure empathy in typically developing children and adolescents aged 10 and older.

## Introduction

Empathy, the ability to share and understand emotional states of others (Batson et al., 1987), has a large impact on how people act in social situations. Some people get affected by emotionally charged situations, which might for example result in showing helping behavior, whereas others are not seized with emotion at all (Jolliffe and Farrington, 2006). Previous studies have demonstrated that children with higher levels of empathy are generally better able to regulate their emotions, show less aggression, and act in a more prosocial way (Mehrabian and Epstein, 1972; Eisenberg, 2000; Meuwese et al., 2015). More specifically, higher affective empathy (i.e., sharing an emotional state) predicts constructive conflict resolution when encountering problems with friends (De Wied et al., 2007). Along the same lines, cognitive empathy (i.e., understanding emotional states of others) predicts higher quality friendships involving mutual reciprocity and stability (Chakrabarti and Baron-Cohen, 2006; Soenens et al., 2007). Overall, empathy is important for bonding with primary caregivers, friends, and other eminent people (Knafo et al., 2008). The lack of empathy has also been related to the development of problem behaviors. For instance, cognitive empathy without the affective component is related to higher levels of bullying (Jolliffe and Farrington, 2006). In a similar vein, affective empathy can hamper rather than strengthen the relationship when the person lacks the ability to support the other person in distress (Pouw et al., 2013). It is therefore crucial to reliably assess empathy in children and adolescents. This study presents the development and validation of an empathy self-report questionnaire for children and adolescents, which disentangles three components of empathy: affective empathy, cognitive empathy, and intention to comfort.

Up until now, validated questionnaires that have been most commonly used with child and adolescent populations are the Interpersonal Reactivity Index (IRI; Davis, 1980) and the Index of Empathy for Children and Adolescents (IECA; Bryant, 1982). However, the validation of both questionnaires has been done with young adults; yet recently the IRI has also been validated in adolescents (Hawk et al., 2013). The IRI consists of four subscales where only the subscales Empathic concern, Fantasy, and Perspective taking aim to measure empathy; the fourth Personal Distress subscale assesses arousal in response to discomfort of others, which is more linked to emotion regulation than to empathy (Cliffordson, 2002). The items of the IECA (Bryant, 1982) originate from the Questionnaire Measure of Emotional Empathy (QMEE; Mehrabian and Epstein, 1972) and aim to measure arousal in response to situations and are more focused on experienced emotions rather than on general affective empathy (Baron-Cohen and Wheelwright, 2004; Jolliffe and Farrington, 2006). With the Empathy Questionnaire for Children and Adolescents (EmQue-CA) we aimed to assess cognitive empathy defined as ‘understanding feelings of others’ more thoroughly and provide a scale that measures both affective and cognitive empathy in a way that is suitable for children and adolescents.

Moreover, existing empathy questionnaires are mainly focused on assessing sharing feelings of others (Mehrabian and Epstein, 1972; Davis, 1983; Jolliffe and Farrington, 2006; Lietz et al., 2011; Hawk et al., 2013), and understanding these feelings (Hogan, 1969; Davis, 1983; Baron-Cohen and Wheelwright, 2004; Jolliffe and Farrington, 2006; Lietz et al., 2011; Hawk et al., 2013). Hereby, the corresponding tendency to behave in supportive ways toward the person in distress has so far been underrepresented (Rieffe et al., 2010). Considering the crucial importance of empathic care for the well-being of people one interacts with, attention to comfort should receive more attention (Batson, 2009; Decety et al., 2016). Therefore, we designed an empathy questionnaire for children and young adolescents (EmQue-CA), suitable for children from the age of 10, that specifically focuses on these three aspects of empathy: (1) *affective empathy*: a scale that measures the extent to which the child/adolescent feels for the emotional state of the suffering person, (2) *cognitive empathy*: a scale that measures the extent to which the child/adolescent understands why the other person is in distress, and (3) *intention to comfort*: a scale that measures the extent to which the child/adolescent is inclined to actually help or support the suffering person.

The current study aimed to examine the construct, convergent, and concurrent validity of the EmQue-CA. The initial questionnaire of 21 items was assessed in a large sample of 10–15-years-old participants (*N* = 1250). To test the intended three-factor structure of the questionnaire, the total sample was divided in two, where a Principal Component Analysis (PCA) was conducted on the first half and a Confirmatory Factor Analysis (CFA) was conducted on the second half of the sample. For concurrent validity, the associations of the EmQue-CA scales with related concepts were examined. We expected that adolescents who scored high on affective empathy had more awareness of what happened in their body following emotional situations. Understanding what is happening in your own body makes it easier to distinguish your emotions from those of someone else, which is expected to promote feelings of empathy and to prevent personal distress (Rieffe and De Rooij, 2012). Additionally, we expected that adolescents scoring high on cognitive empathy and intention to comfort would be more focused on others’ emotions (Grynberg and Pollatos, 2015). Concerning interpersonal functioning, we expected that adolescents who score high on affective empathy would bully less (Stavrinides et al., 2010) and have better friendship qualities as these adolescents feel for another person in distress, which is a crucial contributor to high friendship quality (Berndt, 2002). Moreover, higher friendship quality was expected to be related to cognitive empathy, as understanding why someone feels distressed is important for maintaining positive social relationships.

There is a controversy in the literature suggesting on the one hand that bullies score high on cognitive empathy, which makes them the so-called ‘cold-blooded intelligent bullies’ (Sutton et al., 1999; Rieffe et al., 2012). This was also supported by a study performed by Cheng et al. (2012), who demonstrated that adolescents scoring high on callous-unemotional traits (limited prosocial emotions) were perfectly able to understand and evaluate emotions of others but who showed specific deficits in affective empathy. On the other hand, literature is suggesting that adolescents high on cognitive empathy bully less because of a better understanding of the distress that bullying causes for the other person (Kokkinos and Kipritsi, 2011). This study therefore also assessed the relation between bullying and cognitive empathy.

The final aim of the current study was to examine empathy across childhood and adolescence by studying age differences. Additionally, we explored the role of gender in age-related changes in empathy. With regard to girls, we expected affective empathy to remain stable or show a slight increase, whereas we expected cognitive empathy to clearly increase toward mid-adolescence (Van der Graaff et al., 2014). The latter finding was also supported by Taylor et al. (2013). In boys, we expected a decrease in affective empathy toward mid-adolescence (Van der Graaff et al., 2014). This is in line with our knowledge that testosterone levels in boys increase during adolescence, which could lead to lower levels of empathy (Hermans et al., 2006). Based on previous findings on cognitive empathy in boys, we expected to find a developmental increase in boys (Taylor et al., 2013; Van der Graaff et al., 2014). Finally, we hypothesized to find an increase in intention to comfort with age, which was expected to be stronger in girls (Güroğlu et al., 2014; Klein et al., 2015).

Besides hormonal changes, the social environment could have a large impact on age-related changes in empathy too. The gender identification hypothesis refers to pressure from the social environment (including parents, peers, and social media) to conform to cultural norms with regard to performing specific gender roles (Hill and Lynch, 1983). Hill and Lynch (1983) reported that girls indicated to be highly concerned with their looks and interpersonal relationships, whereas boys reported to be occupied with becoming independent, and to compete with others. The combination of higher testosterone levels in boys (Büttler et al., 2016), and the social environment expecting a more competitive attitude, could be related to lower empathy levels in boys compared to girls. In general, we hypothesized that empathy would show an age-related increase, though with prudence since previous studies have shown inconsistencies (Davis and Franzoi, 1991; Eisenberg et al., 2005; Hermans et al., 2006).

## Materials and Methods

### Participants

Thousand two hundred and fifty children and adolescents aged 10–15 participated in the current study (*M* age = 13.25; *SD* = 1.67; 50% girls; see **Table 1** for the descriptive statistics). The complete sample is a combination of five subsamples, which did not differ in study variables or demographic composition (see Supplementary Table S1 for an overview). To determine ethnicity, the participants were asked to report their own country of origin, the country of origin of their father, and the country of origin of their mother (see **Table 2** for an overview). To get a clear overview, we classified ethnic background by continent (the Netherlands excepted). All participants were recruited from local elementary and high schools with different levels of education, including preparatory schools for vocational secondary education and university.

**Table 1:** Sample sizes, gender distribution (%), mean age and standard deviation (SD) in years of the participants from six age groups.

	10 years	11 years	12 years	13 years	14 years	15 years
Number of participants	157	128	193	278	279	215
Female (%)	78 (50)	69 (54)	86 (45)	144 (52)	143 (51)	106 (49)
Mean age	10.30	11.26	12.59	13.44	14.39	15.47
*SD* age	0.32	0.30	0.26	0.29	0.28	0.27

*The Chi square analyses indicated that gender distribution did not differ across the age groups (X^*2*^ = 34.65, p = 0.60).*

**Table 2:** Ethnicity of participants, and their parents classified by continent.

	Country of origin child/adolescent (N)	Country of origin father (N)	Country of origin mother (N)
The Netherlands	839	508	471
Other European country	38	33	53
North America	7	5	14
South America	31	148	159
Africa	12	142	139
Asia	43	162	161
Australia	3	2	2
Missing data	277	250	251

### Procedures

Data collection took place at school, where participants were given classical instructions. The experimenters emphasized that participation was entirely anonymous and voluntary and that there were no right or wrong answers. Participants could ask questions at any time during the testing session, which was part of a larger session that lasted 45 min on average. The university ethical committee approved the procedures and questionnaires, and both schools and parents provided written consent for participation. Moreover, all participants were treated in accordance with the Declaration of Helsinki. Only healthy participants, with no history of neurological or psychiatric disorders, were included.

### Measures

The initial version of the EmQue-CA consisted of 21 items generated by the second author and had three scales: (1) *affective empathy* (nine items; e.g., ‘When a friend is upset, I feel upset too’) measuring the extent to which one feels for the emotion of another person, (2) *cognitive empathy* (six items; e.g., ‘If a friend cries, I often understand what has happened’) measuring the extent to which one understands why another person is in distress, and (3) *intention to comfort* (six items; e.g., ‘If a friend is sad, I want to do something to make it better’) measuring the extent to which one is also inclined to actually help or support a person in need. Participants were asked to rate to what extent the description was true for them on a 3-point scale: (1) not true, (2) somewhat true, and (3) true (Pouw et al., 2013). All questions were (re)scored such that higher scores reflect higher empathy. Mean scores were calculated per scale. See **Table 3** for Cronbach’s alphas.

**Table 3:** Construct consistencies of the three scales of the Empathy Questionnaire for children and adolescents (EmQue-CA).

	Number of items	*N*	Cronbach’s Alpha	Inter-item correlation	Mean (SD) boys	Mean (SD) girls
**EmQue-CA**						
Affective empathy^∗∗∗^	6	1250	0.70	0.28	1.60 (0.36)	2.04 (0.40)
Cognitive empathy^∗∗∗^	3	1250	0.70	0.44	2.17 (0.50)	2.36 (0.49)
Intention to comfort^∗∗∗^	5	1250	0.74	0.36	2.45 (0.44)	2.66 (0.36)
**IRI**						
Affective empathy^∗∗∗^	6	126	0.74	0.33	1.87 (0.74)	2.53 (0.57)
Cognitive empathy^∗∗∗^	6	1098	0.69	0.27	2.43 (0.63)	2.61 (0.63)
**EAQ**						
Differentiating emotions	7	117	0.75	0.30	2.39 (0.45)	2.34 (0.41)
Bodily awareness of emotions^∗∗^	5	117	0.61	0.24	1.93 (0.44)	1.68 (0.42)
Attending to others’ emotions^∗∗∗^	5	117	0.59	0.22	2.37 (0.40)	2.62 (0.33)
Friendship quality^∗∗∗^	17	862	0.90	0.37	3.53 (0.63)	4.05 (0.59)
Bullying^∗∗∗^	6	907	0.80	0.42	0.94 (0.58)	0.66 (0.49)

*Significant gender differences are indicated with an asterisk: ^∗^*p* < 0.05, ^∗∗^*p* < 0.01, ^∗∗∗^*p* < 0.001.*

#### Interpersonal Reactivity Index

Two scales of the IRI (Davis, 1983) were obtained from different subsamples of this study: empathic concern (e.g., ‘I am often quite touched by things that I see happen’; *N* = 160; hereafter referred to as *affective empathy*), and perspective taking (e.g., ‘I believe that there are two sides to every question and I try to look at them both’; *N* = 1098; hereafter referred to as *cognitive empathy*). Both scales contained six items; each item was rated on a 5-point Likert scale with (0) completely untrue, (1) not quite true, (2) in between, (3) quite true and (4) completely true. All questions were (re) scored such that higher scores reflected higher empathy. Mean scores were calculated per scale. See **Table 3** for Cronbach’s alphas.

#### Emotion Awareness Questionnaire

Three scales of the well validated EAQ were included in a subsample of this study (Rieffe et al., 2008; *N* = 117): *differentiating emotions* (seven items, of which seven recoded; e.g., ‘Sometimes, I feel upset and I have no idea why’), *bodily awareness of emotions* (five items, of which four recoded; e.g., ‘When I feel upset, I can also feel it in my body’) and *attending to others’ emotions* (five items, of which two recoded; e.g., ‘I don’t care about how my friends are feeling inside’). All items were answered on a 3-point scale with (1) not true, (2) sometimes true, (3) often true. Mean scores were calculated per scale and higher scores indicated better abilities in differentiating own emotions, higher bodily arousal in response to own emotions, and higher attendance to others’ emotional states. See **Table 3** for Cronbach’s alphas.

#### Friendship Quality Scale (FQS)

We used an adapted version of the FQS (Bukowski et al., 1994; *N* = 862) containing 28 items assessing *positive* (such as closeness, companionship, and security) and *negative friendship quality* (such as conflict and imbalance). Participants were asked to indicate how much each item was true for their relationship with their best friend on a 5-point Likert scale ranging from (1) not true to (5) completely true. Considering the strong links between positive friendship quality and empathy (Meuwese et al., 2015), we included here the positive *friendship quality* factor with a total of 17 items (e.g., ‘My friend and I help each other’). Mean scores were calculated and higher scores indicated higher relationship quality. See **Table 3** for Cronbach’s alphas.

#### Olweus Bully/Victim Questionnaire (OB/VQ)

Self-reported *bullying* was assessed using the well-validated OB/VQ (Olweus, 1986; *N* = 907). Participants were asked to indicate how often they engaged in different acts of bullying in the last 6 months on a 5-point scale with answers varying from (0) I haven’t bullied other children to (4) several times a week. Mean scores of the six *bullying* items were used; higher scores indicated more engagement in bullying behavior. See **Table 3** for Cronbach’s alphas.

### Statistical Analyses

In order to assess the construct validity of the EmQue-CA, we randomly split the total sample in two. The first half of the sample was used to examine the factor structure by conducting Principal component analyses (PCA), using SPSS version 23. To determine the number of factors to be retained parallel analysis was used (Horn, 1965). In parallel analysis, multiple datasets are randomly generated that equal the original dataset in terms of sample size and number of items. Eigenvalues obtained from the sample data are compared to eigenvalues obtained from the random datasets. Eigenvalues of the sample data bigger than their random counterpart are retained, ensuring that a factor explains more variance than is expected based on chance. In this study, 1000 random data sets were generated. A factor loading of at least >0.40 was required on their key factor. Oblimin rotation with Kaiser normalization was used since factors were assumed to be correlated.

On the second half of the sample a Confirmatory factor analysis was conducted to test the final model as derived by the previous PCA, using EQS version 6.3. All items were hypothesized to load on their key factor; no cross-loadings or error covariances were allowed. Goodness of fit was evaluated using the following cut-off criteria: CFI > 0.90, RMSEA < 0.08, SRMR < 0.08 (Hu and Bentler, 1995, 1999).

Furthermore, the inter-factor correlations were computed to demonstrate diversity between the scales, and Cronbach’s alphas were computed to examine construct consistencies. Next, we examined the associations between the scales of the EmQue-CA with the scales of the IRI to determine convergent validity. The concurrent validity of the EmQue-CA was also tested, for which we examined the associations between the EmQue-CA scales with emotion awareness, friendship quality, and bullying behavior. Finally, we applied Fisher r-to-Z transformation to calculate a value of Z to assess the significant differences between boys and girls for the correlations between the scales of the EmQue-CA with the scales of the IRI, EAQ, FQS, OB/VQ, and age.

## Results

### Construct Validity

#### Principal Component Analysis

A PCA on all 21 items was performed using the first half of the sample. The Kaiser-Meyer-Olkin (KMO) measure was 0.82, which indicated adequate sampling. In addition, KMO values for individual items had sampling adequacy coefficients ranging from 0.51 to 0.90, which are all above the acceptable limit of 0.5 (Field, 2013). Bartlett’s test of sphericity was significant (*χ*^2^= 2596.94, *df* = 210, *p* < 0.001), confirming suitability of the data for PCA.

To extract the suitable number of factors, an initial PCA analysis was conducted. The parallel analysis indicated that four factors should be retained. The four factors together explained 43.38% of the total variance. In the subsequent four-factor PCA with rotation, four items were excluded since loadings on all factors were <0.40 (i.e., “If a friend is laughing, I also laugh,” “I understand that a friend is ashamed when he/she has done something wrong,” “I understand that a friend is proud when he/she has done something good,” “I enjoy giving a friend a gift”). Exploring the content of the items within the factors it was noted that three factors clearly represented the three hypothesized theoretical constructs: (1) affective empathy (six items), cognitive empathy (three items), and intention to comfort (five items). The fourth factor consisted of three theoretically unrelated items (i.e., “If a friend is sad, I also feel sad,” “When a friend cries, I feel mostly nothing,” “I often don’t understand why someone gets angry”) and were therefore removed from further analysis, resulting in retaining three factors.

Subsequently, another PCA was performed with the 14 final items. The overall KMO measure (0.80), the KMO values for individual items (range = 0.74–0.89), and Bartlett’s Test of sphericity (*χ*^2^= 1839.64, *df* = 91, *p* < 0.001) all indicated that data was adequate for PCA. Parallel analysis indicated a 3-factor solution. The three factors combined explained 48.35% of the total variance. Following rotation, all items loaded on the intended factor; the following analyses were thus performed with the newly formed three scales. See **Table 4** for a complete list of all items and factor loadings.

**Table 4:** Factor Loadings for the Principal Component Analysis and the Confirmatory Factor Analysis with the three factor model of the Empathy Questionnaire for Children and Adolescents (EmQue-CA).

		Principal Component Analysis			Confirmatory Factor Analysis		
		Affective empathy	Cognitive empathy	Intention to comfort	Affective empathy	Cognitive empathy	Intention to comfort
1.1	If my mother is happy, I also feel happy	0.668			0.643		
1.2	I often feel sad when I watch a sad movie	0.633			0.624		
1.3	When a friend is upset, I feel upset too	0.588			0.450		
1.4	When a friend cries, I cry myself	0.775			0.622		
1.5	If someone in my family is sad, I feel really bad	0.473			0.530		
1.6	I feel awful when two people quarrel	0.469			0.455		
2.1	When a friend is angry, I tend to know why		0.848			0.640	
2.2	If a friend is sad, I understand mostly why		0.827			0.861	
2.3	If a friend cries, I often understand what has happened		0.560			0.540	
3.1	If a friend is sad, I like to comfort him			0.490			0.666
3.2	I would like to help when a friend gets angry			0.723			0.646
3.3	If a friend has an argument, I try to help			0.447			0.549
3.4	I want everyone to feel good			0.724			0.454
3.5	If a friend is sad, I want to do something to make it better			0.807			0.714

*Only factor loadings > 0.30 are printed in this table.*

#### Confirmatory Factor Analysis

The final model obtained with PCA was directly tested with a CFA, using the second half of the sample. Given that Mardia’s normalized coefficient (13.21) indicated substantial multivariate kurtosis, analyses were based on the robust Satorra-Bentler *χ*^2^ statistic. The results indicated good fit to the data [S-B*χ*^2^ (df = 74, *N* = 625) = 230.59, *p* < 0.001; SRMR = 0.055; CFI = 0.912; RMSEA = 0.058, 90% C.I. = 0.050, 0.067]. Standardized factor loadings ranged from 0.450 to 0.861. Thus, the three-factor model based on the final set of 14 items was retained (see **Table 4**).

#### Reliability Analyses, Convergent Validity and Links with Gender

Cronbach’s alphas of the three scales of the EmQue-CA indicated good internal consistencies (Nunnally, 1978) for the scales: *affective empathy* 0.70, *cognitive empathy* 0.70, and *intention to comfort* 0.74 (see **Table 3**). The mean scores in **Table 3** show that girls scored higher on *affective empathy, cognitive empathy*, and *intention to comfort*. Correlations between all three scales were significant, but still below 0.38 indicating that there was no collinearity (see **Table 5**). The correlations remained significant when analyzed separately per age group (see **Table 1** for more information about the age groups). Additionally, the *affective* and *cognitive empathy* scales of the EmQue-CA correlated positively with the respective IRI scales (see **Table 5**). **Table 3** also presents the means of all included constructs separately for boys and girls. Boys and girls differed significantly on all measures, except for differentiating emotions. Girls scored higher on all empathy measures, attending to others’ emotions and friendship quality; boys reported significantly higher levels of bodily awareness of emotions and bullying than girls.

**Table 5:** Correlations between the scales of the EmQue-CA and the scales of the IRI, EAQ, friendship quality, and bullying.

	Affective empathy	Cognitive empathy	Intention to comfort
**EmQue-CA**			
Affective empathy	1		
Cognitive empathy	0.30***	1	
Intention to comfort	0.38**	0.35**	1
**IRI**			
Affective empathy	0.52***	0.28***	0.33***
Cognitive empathy	0.31***	0.30***	0.40***
**EAQ**			
Differentiating emotions	-0.31***	-0.06	-0.03
Bodily awareness of emotions	-0.36***	-0.01	-0.19*
Attending to others’ emotions	0.29**	0.42***	0.15
Friendship quality	0.35***	0.30***	0.39***
Bullying	-0.17***	-0.18***	-0.26***

*^∗^*p* ≤ 0.05, ^∗∗^*p* ≤ 0.01, ^∗∗∗^*p* ≤ 0.001. All correlations remained significant when corrected for age.*

### Concurrent Validity and Links with Gender

To examine the concurrent validity of the EmQue-CA, we tested the relationship between the EmQue-CA scales and related constructs (see **Table 5**). The results showed a negative correlation between *affective empathy* and *differentiating emotions*, and a negative correlation between *affective empathy* and *bodily awareness of emotions* and *intention to comfort* and *bodily awareness of emotions*. *Affective empathy* and *cognitive empathy* were both related positively to higher scores on the EAQ scale *attending to others’ emotions.* When we examined the relationships of the EmQue-CA scales with indices of social functioning, we found that *friendship quality* correlated positively with *affective empathy*, and *intention to comfort*. *Bullying*, on the other hand, correlated negatively with all three scales of the EmQue-CA. All correlations remained significant after controlling for age.

To test for gender differences regarding the strengths of the relationships (i.e., the strength of the correlations) between scales of the EmQue-CA and other variables in this study, we applied Fisher r-to-Z transformation. The correlation between *cognitive empathy* as measured by the EmQue-CA and by the IRI showed a difference between boys and girls, with girls showing a stronger positive correlation between the two scales than boys (*Z* = -2.78; *p* < 0.001). Additionally, results showed a significant gender difference in the correlation between *affective empathy* and *differentiating emotions* (*Z* = -2.71; *p* < 0.01), and between *intention to comfort* and *attending to others’ emotions* (*Z* = 2.91; *p* < 0.01); in both cases, the correlations were only significant for boys.

### Gender and Age Associations

We tested for gender differences in the correlations of the EmQue-CA scales and age by applying Fisher r-to-Z transformation to calculate a value of *z*. These analyses showed a significant gender difference for the link between *affective empathy* and age (*z* = -4.33; *p* < 0.001), *cognitive empathy* and age (*z* = -3.47; *p* < 0.001), and *intention to comfort* and age (*z* = -2.06; *p* < 0.05). Girls showed a positive relationship between age and *affective empathy* [*r*(626) = 0.14, *p* < 0.001] and *cognitive empathy* [*r*(626) = 0.10, *p* < 0.05], whereas there was a negative relationship for boys between age and *affective empathy* [*r*(626) = -0.10, *p* < 0.01] and *cognitive empathy* [*r*(626) = -0.10, *p* < 0.05]. Moreover, boys showed a negative correlation between age and *intention to comfort* [*r*(624) = -0.17, *p* < 0.001]; an effect that was absent in girls.

## Discussion

In this study, we examined the construct, convergent, and concurrent validity of the EmQue-CA, a newly developed empathy questionnaire for children and adolescents. The EmQue-CA aims to assess affective- and cognitive empathy and the tendency to care for another’s welfare as a result of empathy. The outcomes of this study confirm a three-factor structure within the EmQue-CA. Moreover, the three identified scales show good internal consistencies, as well as the expected relationships with another validated empathy measure (i.e., the IRI), and related constructs of emotion awareness and differentiation (as assessed by the EAQ), and social functioning (i.e., friendship quality and bullying).

The negative correlations of the affective empathy scale and the intention to comfort scale of the EmQue-CA with the scale bodily awareness of emotions of the EAQ were as expected. Children and adolescents who focused more on other people’s distress (as assessed by the affective empathy scale) and who had a stronger tendency to help others (as assessed by the intention to comfort scale) were more focused on their own internal state as assessed by bodily awareness of emotions. This is in line with prior findings showing that being aware of your own emotions and the emotions of others is related to reporting less depressive symptoms (Rieffe and De Rooij, 2012). Overall, our results emphasize the importance of bodily awareness and the positive effect of paying sufficient attention to the outside world and other people’s mental states. Cognitive empathy, however, did not relate to bodily awareness, suggesting that being attentive to bodily changes is not crucial for understanding others’ emotional states. Additionally, children and adolescents who were more affected by other people’s distress, as assessed by the affective empathy scale, were better able to understand why they were feeling, for example, angry or upset (as assessed by differentiating emotions). Yet, follow up comparisons showed that this negative relationship between affective empathy and differentiating emotions was only significant in boys, suggesting that internal feelings of girls did not seem to influence their ability to show concern for others. A suggestion that could be explained by the gender identification hypothesis stating that girls are socialized into specific gender roles (e.g., knowing how to express and communicate about emotions) by their environment, especially across adolescence (Hill and Lynch, 1983).

This could be explained by gender differences in affective empathy as proved by several experiments using different neurophysiological measures (see Christove-Moore et al., 2014 for a review). Overall, they conclude that “emotional contagion” in females is higher and less discriminative –females are equally empathic toward males and females– whereas males are predominantly empathic toward females. Since this difference was specifically found in affective empathy, this could also explain why in our study differentiating emotions did not relate to cognitive empathy and intention to comfort. In future studies, the exact mechanism should be examined more extensively.

In order to sense the feelings of another person, one needs to overcome the idiosyncratic focus and be able to focus on the other person’s feelings instead. This was captured by the scale attending to others’ emotions, which was related to all three scales. In other words, in order to feel, understand and act in a prosocial way, one needs to be able to attend to the emotions of others. However, the positive relationship between attending to others’ emotions and intention to comfort was again only significant in boys. This result could indicate that the willingness to help others is the main reason for boys to attend to others’, whereas girls attend to others’ emotions without feeling the urge to be supportive. This should be examined in more detail in future studies; more insight in this gender difference can be highly informative for understanding the development of empathy and prosocial behavior.

To further unravel the specific role of empathy and intention to comfort in social interactions during childhood and adolescence, we examined how the scales related to both positive and negative aspects of social relationships. The positive link between friendship quality and all three scales is in line with our expectations and confirms the importance of empathy for social interactions irrespective of gender (Soenens et al., 2007; Allemand et al., 2014). Due to inconsistent findings on the link between empathy and bullying behavior in prior studies, we did not have *a priori* expectations. Our results show that high levels of affective empathy, cognitive empathy and intention to comfort were associated with lower levels of bullying behavior. Importantly, bullying behavior was related to all three scales, supporting the protective role of empathy in offensive behaviors.

Examining age-related changes in empathy, our results showed an increase in affective empathy and cognitive empathy in girls, and a decrease in affective empathy, cognitive empathy, and intention to comfort in boys across childhood and early adolescence. The fact that we found contradictory findings for males and females regarding the relationship between empathy and age could be explained by the gender identification hypothesis (Hill and Lynch, 1983). An increase in affective and cognitive empathy with age in girls is in line with gender specific socialization processes where the social environment expects girls to attach importance to interpersonal relations. These interpersonal relationships become more and more important during adolescence, which could be related to higher self-reported empathy. Boys on the other hand are stimulated to focus on high achievements, whereby a competitive attitude is highly valued (Hill and Lynch, 1983). Since adolescence is important for developing independency and individuality, this gender role could result in decreased self-reported empathy in boys. Moreover, a prior study by Hermans et al. (2006) showed that high baseline testosterone levels are related to low empathy scores. Considering the fact that boys tend to have increased testosterone levels, specifically during adolescence, this could be a possible explanation for the negative relationship between all 3 empathy scales.

Apart from the age-related effects, the finding that self-reports of affective empathy, cognitive empathy, and intention to comfort were higher in girls than in boys was in accordance with previous findings, which indicate that girls more often tend to get affected by and share emotions with friends and other important people (Taylor et al., 2013). However, the age-related increase in affective empathy deviates from the study performed by Van der Graaff et al. (2014) who found that affective empathy was stable in girls from age 13 onward. Allemand et al. (2014) performed a longitudinal study including 10–16 year olds at the first time-point to test whether empathy would be predictive for social skills around the age of 35. Their results showed that girls scored generally higher than boys. However, they also found individual differences in the developmental trajectory of empathy in both boys and girls. Some participants showed a decrease in empathy – predictive for worse social skills later in life –, whereas others showed an increase in empathy that was indicative for higher social skills in adulthood. Overall, this emphasizes the importance of investigating the role of individual differences beyond gender differences. Moreover, we also found a developmental decrease in intention to comfort in boys. Similar effects were reported by Meuwese et al. (2015) who demonstrated an age-related decrease in equity preference specifically in boys. This decrease could be linked to a decrease in prosocial behavior, especially when being fair (i.e., a form of prosocial behavior) is costly. However, it should be noted that the differences in mean levels of intention to comfort in the different age groups are small and have to be interpreted with caution. Therefore, longitudinal studies are necessary to confirm these age trends.

Additionally, the issue of self-reports should also be taken into account since social desirability, response style, and social expectations could have an effect on the sex differences we found. Therefore, future studies should also investigate links between the EmQue-CA and parent-, peer-, or teacher-reports of other measures.

Empathy is an important capacity for showing adaptive and efficient behavior in social interactions, emphasizing the importance of investigating this more thoroughly. Especially in less well developing populations (such as Autism or Oppositional Defiant Disorder) divergent social behaviors may be related to delayed or lacking development of one or more components of empathy. For example, several studies have denoted the possibility of higher levels of affective empathy combined with lower levels of cognitive empathy in children and adolescents with Autism Spectrum Disorder (ASD) (Deschamps et al., 2014; Pijper et al., 2016). The discrepancy between these two components of empathy might create tension for these children and adolescents who do not know how to respond adaptively when experiencing affective arousal (Pouw et al., 2013). In contrast, the rule-breaking behavior of children and adolescents diagnosed with Oppositional Defiant Disorder (ODD) or Conduct disorder (CD) could be due to lower levels of affective empathy, which in turn could hinder them in forming and maintaining friendships with peers (Taylor et al., 2013).

Taken together, the EmQue-CA presents a significant contribution to the literature on empathy and intention to comfort in children and adolescents. Specifically, the scale assessing intention to comfort presents a valuable addition to existing questionnaires measuring empathy. Examining intention to comfort is particularly important during childhood and adolescence, as prosocial behavior is crucial for adaptive socio-emotional functioning. We demonstrate here that the three scales of the EmQue-CA show differential links with age and gender and thus contribute uniquely to the assessment of empathy. Future studies should further employ the EmQue-CA to unravel how empathy and intention to comfort contribute to psychosocial functioning, as these concepts are of great importance in forming and maintaining social relationships across childhood and adolescence.

## Ethics Statement

This study was carried out in accordance with the recommendations of APA guidelines, with informed consent from all subjects. All subjects gave written informed consent in accordance with the Declaration of Helsinki. The protocol was approved by the Ethics Committee of Leiden University.

## Author Contributions

Substantial contributions to the conception or design of the work; or the acquisition, analysis, or interpretation of data for the work: SO, CR, EB, and BG. Drafting the work or revising it critically for important intellectual content: SO, CR, EC, and BG. Final approval of the version to be published: SO, CR, EB, EC, and BG. Agreement to be accountable for all aspects of the work in ensuring that questions related to the accuracy or integrity of any part of the work are appropriately investigated and resolved: SO, CR, EB, EC, and BG.

## Conflict of Interest Statement

The authors declare that the research was conducted in the absence of any commercial or financial relationships that could be construed as a potential conflict of interest.
