# Supplementary material for: Assessing Empathy across Childhood and Adolescence: Validation of the Empathy Questionnaire for Children and Adolescents (EmQue-CA)
Source: Front Psychol. 2017 May 29;8:870. doi: 10.3389/fpsyg.2017.00870 (PMC5447078; doi:10.3389/fpsyg.2017.00870)
Supplement: Supplementary file 1 [file Table_1.docx]

TABLE S1 | Sample sizes (separately for each questionnaire), gender distribution (%), mean age and standard deviation (SD) in years for each individual subsample.

|  | Subsample 1 | Subsample 2 | Subsample 3 | Subsample 4 | Subsample 5 |
| --- | --- | --- | --- | --- | --- |
| N | 973 | 78 | 48 | 117 | 34 |
| Females (%) | 477 (49) | 50 (64) | 20 (42) | 61 (52) | 17 (50) |
| Mean age (SD) | 13.75 (1.41) | 10.65 (.75) | 11.90 (1.67) | 11.93 (1.20) | 11.35 (.78) |
|  |  |  |  |  |  |
| EmQue-CA |  |  |  |  |  |
| Affective empathy (N) | 973 | 78 | 48 | 117 | 34 |
| Cognitive empathy (N) | 973 | 78 | 48 | 117 | 34 |
| Intention to comfort (N) | 973 | 78 | 48 | 117 | 34 |
|  |  |  |  |  |  |
| IRI |  |  |  |  |  |
| Affective empathy (N) | 0 | 78 | 48 | 0 | 0 |
| Cognitive empathy (N) | 973 | 78 | 48 | 0 | 34 |
|  |  |  |  |  |  |
| EAQ |  |  |  |  |  |
| Differentiating emotions (N) | 0 | 0 | 0 | 117 | 0 |
| Bodily awareness of emotions (N) | 0 | 0 | 0 | 117 | 0 |
| Attending to others’ emotions (N) | 0 | 0 | 0 | 117 | 0 |
|  |  |  |  |  |  |
| Friendship quality (N) | 862 | 0 | 0 | 0 | 0 |
|  |  |  |  |  |  |
| Bullying (N) | 966 | 0 | 0 | 0 | 0 |
